# Rewealthization in twenty-first century Western countries: the defining trend of the socioeconomic squeeze of the middle class

**DOI:** 10.1186/s40711-020-00135-6

**Published:** 2021-01-11

**Authors:** Louis Chauvel, Eyal Bar Haim, Anne Hartung, Emily Murphy

**Affiliations:** 1 Department of Sociology, University of Luxembourg, Esch-Belval, Luxembourg; 2 Institute for Research on Socio-Economic Inequality IRSEI, University of Luxembourg, Esch-Belval, Luxembourg; 3 Ben-Gurion University, Beersheba, Israel; 4 STATEC, the National Institute of Statistics and Economic Studies of the Grand Duchy of Luxembourg, Luxembourg City, Luxembourg; 5 SKOPE, University of Oxford, Oxford, UK

**Keywords:** Inequality, Middle-class society, Repatrimonialization, Wealth-to-income ratio

## Abstract

The wealth-to-income ratio (WIR) in many Western countries, particularly in Europe and North America, increased by a factor of two in the last three decades. This represents a defining empirical trend: a rewealthization (from the French *repatrimonialisation)*—or the comeback of (inherited) wealth primacy since the mid-1990s. For the sociology of social stratification, “occupational classes” based on jobs worked must now be understood within a context of wealth-based domination. This paper first illustrates important empirical features of an era of rising WIR. We then outline the theory of rewealthization as a major factor of class transformations in relation to regimes stabilized in the post-WWII industrial area. Compared to the period where wealth became secondary to education and earnings for middle-class lifestyles, rewealthization steepens society's vertical structure; the "olive-shaped" Western society is replaced by a new one where wealth "abundance" at the top masks social reproduction and frustrations below.

**Supplementary Information:**

The online version contains supplementary material available at 10.1186/s40711-020-00135-6.

## Introduction

The dynamics of wealth in the Western world is a central element of the inequality which has ballooned over the last 30 years (Piketty 2014; Wolff 2016; Saez and Zucman 2016; Chauvel et al. 2019). Piketty (2014) has demonstrated this trend on a global level, concluding that “capital is back.” Despite recent research on wealth inequality, the parallel trends pertaining to income *and* wealth are not yet well understood (Jenkins 2009; Kuypers and Marx 2016). While income is more easily measured than wealth, the latter shows unambiguous transformations (Piketty 2014; Saez and Zucman 2016; Chauvel et al. 2019). Analyzing joint relations between income and wealth may help explain the process of a squeezed middle class, and the contradictory nature of progressive tendencies (e.g., educational expansion, gender parities) yoked together with destabilizing tendencies (e.g., legitimation of elitism, middle-class malaise) to differing degrees across welfare regimes (Gornick and Jäntti 2013; Cowell and Van Kerm 2015; Mijs 2019; Semyonov and Lewin-Epstein 2013; Semyonov et al. 2013; Skopek 2015; Chauvel and Hartung 2016; Cowell et al. 2017; Chauvel 2019).

Our main objective in this paper is to assess the relevance of a comeback of wealth as a crucial resource for defining one’s social position in the conceptualization of social class. Relying on a theory of pluralistic middle-class fractions initially developed by Gustav Schmoller and Pierre Bourdieu (Chauvel 2020), we focus here on a successive disruptive factor in stratification dynamics: the comeback of wealth and its major consequences on Western social class systems (Chauvel 2006). The “rewealthization”—or repatrimonialization translated literally from French—presupposes a previous period of “dewealthization” (*dépatrimonialisation* in French). *Dépatrimonialisation* constituted a trend of wealth moderation promoted by the development of strong welfare states. In the post-World War II (WWII) industrial era, the conception of class analysis for Western countries was largely based on the male head of households’ employment, particularly their occupational class, since occupation represented the main structured source of hierarchy among men (and their families): where educational expansion led the competition for jobs and differentials between occupations acted as a primary source of income inequality in society. This in turn grew to encompass women’s entry into a post-industrial workforce and class divisions according to their own labor income. In the wealth-based society promoted by welfare state retrenchments, a newly fashioned hierarchy emerges in the mid-1990s. Over and above a hierarchy of occupations, it is now a hierarchy of ownership which once again becomes a major source of socioeconomic divide.

Contemporary sociologists specialized in social structure and class divisions tend to focus less on income and wealth distributions than on the employment relationship and occupations (typically, Erikson and Goldthorpe 1992, Goldthorpe 2013). In contrast, economic resources in flux (income) and stock (wealth) are the dominant focus in economic studies of the same fields (typically, Piketty 2014). Although many scholars (Bourdieu 1979; Wright 1997; Savage et al. 1992; Savage and Butler 1995; Savage 2015; Liu 2020) conceptualized mixed occupational and resource approaches, social class today is—for both men and women—more a question of what one does than of what one owns. In this context, the role of wealth is more systemic than the role of labor income; as the result of accumulated incomes over a lifetime and as a source of investments in the future, wealth can be transmitted from one generation to the next. Scholars have become increasingly aware over the years (Guo et al. 2018) that class structure is thereby a complex, systemic aggregation of a series of resources garnered from education, occupation, income, wealth, among others. An important aspect of the comeback of wealth in class analysis is to provide a characterization of the “middle class squeeze” problem on alternate and broader grounds than purely employment-based approaches (Wright and Dwyer 2003; Murphy and Oesch 2018; Peugny 2019).

The purpose of this paper is to situate the specific role of wealth in class systems across Western societies and to explore whether rewealthization constitutes a threat for the future stability of the class structure. We first establish the empirical reality of this trend. In a second part of the paper, we argue that this trend is a defining issue of our times with significant consequences for the middle classes. In a third section, we flash back to a former period in the twenty-first century, a period in which the new middle class emerged and rose to dominance as a social group in affluent, wage-based societies. In a fourth section, we contrast this with the more recent dynamics of wealth expansion and lower welfare moderation, which risks destabilizing middle-class lifestyles. We conclude with what rising wealth-to-income ratio may mean for future scenarios and a social morphology of Europe and North America in particular. The current prognosis is bleak: extreme affluence of wealth-based societies could marginalize ever more segments of society, potentially jeopardizing social stability.

## “Wealth is back” as a new social fact

**Fig. 1:**
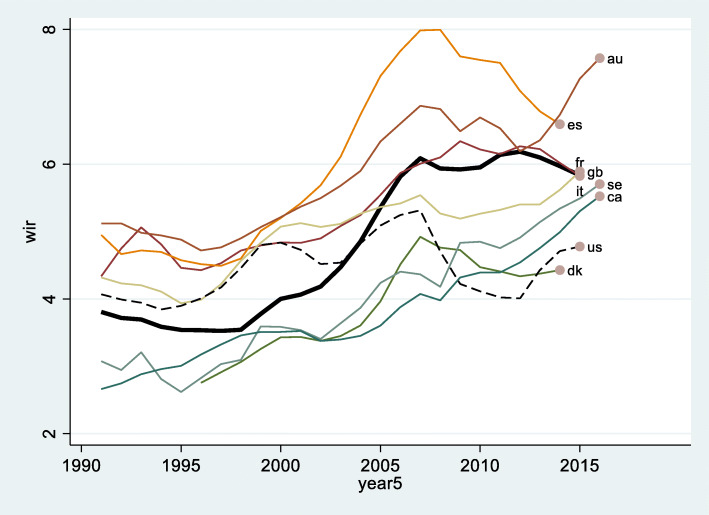
Wealth-to-income ratio in nine Western countries (France: as reference line in bold). Note: Country codes refer respectively to au Australia, ca Canada, dk Denmark, es Spain, fr France, gb Great Britain, it Italy, se Sweden, and us the United States. Source: Authors’ calculations based on the WID, https://wid.world/, see Alvaredo et al. (2017)

Between 1990 and 2020, many Western countries underwent a major transformation in equilibrium between wealth and income. There is a general shift in reliance on flux resources like labor incomes, wages, and premiums arising from economic activity to a reliance on stock resources like wealth, assets, capital, property ownership and rights. Empirical evidence for the occurrence of this rewealthization is found in the striking jump of the formerly balanced aggregated wealth-to-income ratio (WIR) over the 40-year timespan. Wealth-to-income ratios in eight major Western economies are given in Fig. 1, showing the ratio between average per capita net wealth (the total value of cash, housing, bonds, equities, etc., owned by the national economy, minus debts) and the per capita income (the gross domestic product minus fixed capital used in production processes plus the net foreign income earned by residents). Figures are obtained from the World Inequality database (WID) (Alvaredo et al. 2017) through our STATA programming based in the WID command (Blanchet 2017) as developed in the Supplementary information.

The trend of rewealthization varies across countries, but upward trajectories are particularly stark in Europe, notably France, Spain, and Sweden, as well as in North America, notably Canada (Chauvel and Hartung 2016). What we see is a doubling of the WIR over time, though the financial crisis of 2008 hit some countries, such as Spain, Greece or Ireland (Whelan et al, 2017) particularly hard.

Eastern Asian countries, in particular China, experienced similar developments of wealth but in an entirely different context. Western economic growth was, on average, weak in the period 1990–2020 and led to a general stagnation of wage incomes, whereas the rapid economic expansion in China benefited different social classes.

The US dynamic presents an interesting comparison and could constitute an exceptional case due to its relatively stable WIR. However, this is not to say that a trend of rewealthization did not take place in America, but rather that it took on a very concentrated, top-heavy, form. From 1990 to 2015, the *average* accumulation of net assets (wealth) in the US did *not* increase faster than average (labor) incomes. One explanation lies in the accumulation of public deficits, which reduced the net American wealth, as wealth accumulation of the median population became more difficult. Additionally, the US exemplifies a country which experienced a complete gutting of countervailing welfare moderation over the last three decades, and propelled the power grabs of an elite; government protections across the class structure were scrapped as only the top 1% of American society grew their allowances for exorbitant profit from their labor (Huber et al. 2019), but more importantly their wealth.

**Fig. 2:**
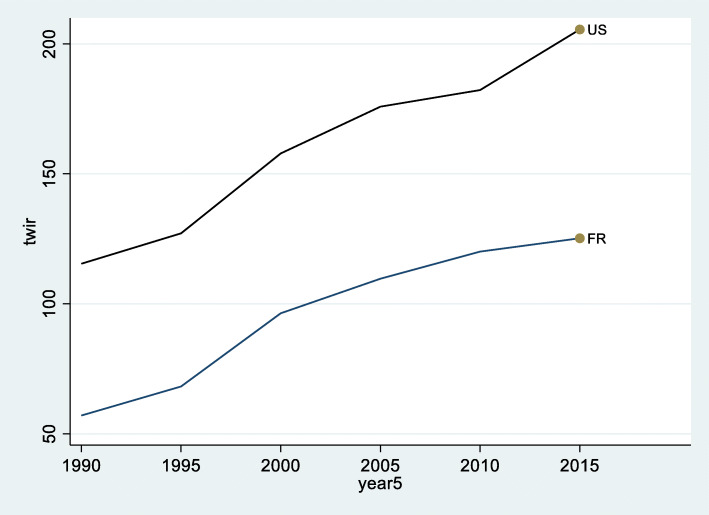
Top wealth (top 1% of the population’s average net wealth) to average income ratio TWIR in the US and France. Source: Authors’ calculations based on the WID, https://wid.world/, see Alvaredo et al. (2017)

Since rewealthization in the US could be a story of exclusively super wealth, we narrow our analysis to the *top* wealth-to-average income ratio (TWIR). The TWIR indicator expresses the average top 1% accumulation of (net) wealth in numbers of years of mean incomes (Fig. 2).

In France, the TWIR ratio rose from 59 to 130 years equivalent of labor income between 1990 and 2015: the average wealth of the top 1% of the wealthiest French residents represented circa 60 years of average income accumulation in the 1990s, and two times more before the Covid-19 era. This is mainly due to the increasing WIR showing the systematic advantage of French owners during that period. In addition, at the very top, the development of earnings among finance managers played an important role for the increase in inequality in France (Godechot 2016). Contrary to the US, CEOs and entertainment superstars did not contribute to the development of inequalities in France.

**Fig. 3:**
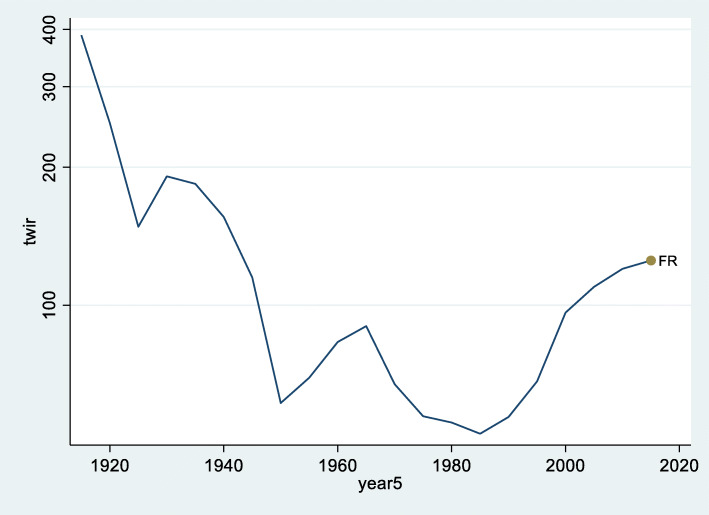
Top wealth (average top 1% population’s net wealth) to average income ratio (TWIR) in France, 1913–2015 (logged *y*-scale). Source: Authors’ calculations based on the WID database, https://wid.world/, see Alvaredo et al. (2017)

In the US, the TWIR jumped from 100 to 171 years. Conversely, this trend cannot be attributed to the WIR (which was stable in the US), but to the expansion of wealth inequality. For a country like France, where the evolution is documented from 1913 onwards, the recent trend of repatrimonialization means a partial regression to pre-World War I (WWI) levels. In 1913, the average top 1% wealth represented 389 years of average income in France. The TWIR decreased to its lowest level of 52 years in 1985. As can be seen in Fig. 3, by 2015, the TWIR had increased again to 127 years. The value of 52 means that the aggregated value of the top 1% population’s wealth is equivalent to 0.52 years of net national income and an increase of 72 years of the TWIR (that soared from 52 years to 127 in France) means this 1% wealthy population now owns 1.27 year of net national income. This is still far from the almost 4 years of GNP that the French top wealthy population of 1913 owned at that time, but it is a clear trend in that direction.

## Relevance of rewealthization for the socioeconomic transformations of our times

Wealth transformations have a major influence on socioeconomic inequality between social classes, but also within apparently homogeneous social classes. Without better knowledge of wealth inequality dynamics, the risk is to accelerate Mathew effects of cumulative advantages and disadvantages due to lack of social investment over the life course (Bonoli et al. 2017). More specifically, the wealth transformation in relation to income dynamics may explain why even in countries with stable income inequality (like France), people can be concerned about economic inequalities: wealth plays a determining role. As such, in many European countries, the Gini indices of income and wealth distribution are stable and yet the increasing WIR results in growing economic inequality: wealth means an increasing number of years of income accumulation, and debts longer periods of reimbursement. The WIR measures this inequality, which deepens when the top-end wealth of large proprietors is compared to the median income of the middle class.

The WIR, initially developed by Stiglitz (1969), is relevant in three dimensions. First, WIR growth measures the change from a wage-based society (relatively egalitarian work compensations as main resource) to a wealth-based society where rewards to work decline (Piketty 2014) and merit is threatened by the prevalence of inherited wealth (Killewald et al. 2017; Ponomarenko 2017). The second dimension is comparative social fact: the doubling of the WIR in many Western countries over the past three decades is a defining issue of our times. The third aspect relates to the distributional structure of income and wealth.

A representation of those differences is exhibited by the “strobiloid” curve (Chauvel 2016), density curves derived from the Pareto (1896) distribution and obtained with kernel density estimation (Van Kerm 2005). The harmonization of scales and surfaces allows comparisons of shapes. The strobiloid opposes the smoothed density curve of the median income (i.e., level of living, defined as the post-tax and transfer net income by consumption unit) to the curve of median net wealth. In more egalitarian countries, the income distribution presents a somewhat “olive shaped” (Li and Zhu 2016) distribution with a rather homogeneous median class, and a small proportion of the population appear at the extremes of affluence or poverty. The strobiloids of wealth are completely different, since the median class of wealth is weak and squeezed between the extreme poor with next to no wealth and the super rich with wealth that sits far above the middle of the wealth distribution.

**Fig. 4:**
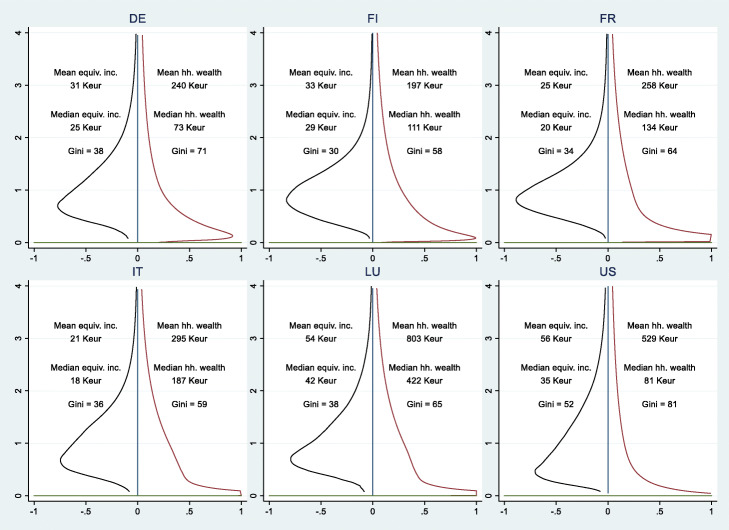
Strobiloids (vertical density curves) of income (left) and wealth (right) in six countries. Note: Country codes refer respectively to DE Germany, FI Finland, FR France, IT Italy, LU Luxembourg, US the United States. The vertical axis represents the medianized levels of equivalized income (left) and household wealth (right). The horizontal axis represents density (surfaces are standardized to 1). The strobiloid is larger when a stronger density of the population is measured at this level of resource. At *Y* = 4, income/wealth is 4 times the median. In countries where a large share of the population has no wealth, the lower part of the strobiloid is truncated. Sources: Incomes from Luxembourg income study (LIS, https://www.lisdatacenter.org/) circa 2012, and wealth from EU-HFCS 2012 for European countries and SCF 2013 for the US. Authors’ calculations and graphics

Figure 4 presents these strobiloids for six Western countries. What each of these country cases illustrates is that density near median wealth is extremely low. By contrast, income inequality varies; income inequality is at a minimum in Finland, with a large population density near to the median, and at a maximum in the US, where the shape is pyramidal. Some Southern European countries, Luxembourg, Poland, and Slovakia, show a slightly stronger density near the wealth median, meaning a model of wealth accumulation in the middle. Unlike income however, wealth does not define a strong middle class in the majority Western countries.

The Gini indices of wealth are relatively stable in Europe and slowly increasing in the US (Chauvel et al. 2018). However, the almost doubled WIR has a major impact on economic inequalities overall due to the shift away from relatively moderate inequalities based on work differentiation (with Gini indices close to .3 in Europe) to much larger inequalities generated through the accumulation of capital (with Gini indices above .6).

Moreover, in societal systems where income inequality is mostly based on work or wages, an individual’s occupation is a measurable, highly visible dimension in the assessment of socioeconomic class definition. By contrast, in the more recent wealth-based society, families’ wealth accumulation is often a less objectively recorded and more veiled source of power and socioeconomic position, demonstrated by the gendered consequences of marital breakdowns where family inheritance concerned (Bessière 2019).

Another consequence of the lack of symmetry between income and wealth (Fig. 4) is in terms of class structure and social dynamics. When inequalities in health, education, and pension are indexed on the lifelong accumulation of households’ savings, like in wealth-based societies before WWI, after neoliberal reforms (Mau 2015) or common in Latin America (Méndez and Gayo 2019), the inter-decile ratio may reach 300 or even more. Under these circumstances, we can witness a dual social structure where two scales of inequalities coexist: the one of income inequality that remains of limited intensity, and the extreme polarization of the wealth distribution where social gradients are potentially boundless. In the US, where the cost of health, education, or old-age savings for seniors’ livelihood are ultimately based on families’ wealth accumulation, the extreme contrasts between the haves and the have-nots are exacerbated. This shift from wage to wealth-based society comes with a massive “sling effect” of wealth inequalities (Chauvel 2016): with the properties of Pareto tails of a distribution, increasing inequalities have a squeezing effect on the middle class, one of extreme acceleration of growth in the *top 1%* of the distribution. These 1% are the segment that enjoyed disproportional gains in newer wealth-based societies.

This trend implicates an important source of distortions—or even tensions—in socioeconomic positions. In the Golden Age of wage-based middle-class societies, when earned incomes were the major source available for consumption or savings, middle classes had a relatively homogeneous self-definition in terms of socioeconomic wellbeing. The relative prestige of neighborhoods, house sizes, and cars were relatively well determined by the purchasing power of households’ earnings. In a wealth-based society, each social stratum, even in the middle class, shows an inner polarization between extremely wealthy members who can afford a standard of living of much richer social groups, and others who must consider the consequences of their limited wage realities (Leicht and Fitzgerald 2013; Temin 2017). For instance, it is common in prestigious universities to see academics of different wealth backgrounds, many live like standard petty-bourgeois, and others own luxury properties in one or several global cities.

An important question the trend of rewealthization raises concerns the consequences of a shift from a strong egalitarian welfare state promoting a dense median wage-earner middle class (typically the corporatist welfare state) to a new wealth-based society where important segments of private and social consumption are available through a market competition of accumulated resources. Where governments have provided little protection from market bidding, such wealth inequalities have been shown to encourage the middle class to take on debt to try and ensure their stability and engage in bidding wars for “positional goods” such as private education and prime housing—one which the wealthy win at greater cost to the rest (Ahlquist and Ansell 2017).

In terms of income, we can suppose a strong homogeneous “median class”, while in terms of wealth, an extreme polarization exists between no-wealth families and the top wealthiest and no homogeneous median class. When “wealth is back,” the disaggregation of the middle class is not necessarily coming from an increase of the income Gini index, but from the growing importance of accumulation measured by the WIR. When the WIR grows like the TWIR (that measures the relative advantage of the richest population to the middle of the income distribution), the same university tuition fee represents an entire lifetime of savings for some middle-class parents, or a casual check amounting to pocket money from wealthy grandparents.

## The emergence of middle-class societies in post-WWII Western countries

In a previous paper on the history of Western middle class in Europe, Chauvel (2009) described the emergence of the idea of middle-class societies in the late nineteenth century with key thinkers of the coming “new middle class,” including Simmel, Schmoller, or Bernstein (see also Charle 2002). An important aspect is the risk of anomic destabilization the middle class can sustain in case of brutal economic recession: Lederer and Marschak (1926) and Geiger (1930) anticipated the “Panic in the middle class” (Geiger 1930) that eventually contributed to the rise of fascist empires. In the post-WWII era, in a context of relatively rapid and egalitarian reconstruction of Western economies, a new model of society emerged: the “middle class society” that resulted in a process of “middlization.”

A more systemic approach than the measurement of inequality can be achieved with a broader conceptualization of “middle class societies.” Drawing on various classical social science works (notably Galbraith), the typical middle-class societies of the industrial times that culminated in the Western world in the eve of the 1980s can be described by seven important parameters—or seven “pillars” of “middlization.”

1. Well above the level of the working class, a new group of wage earners emerges with stable and predictable earnings around the median wage; career stability becomes a norm (or at least a typical model in the public sector and then imitated by large companies in the service sector such as banks, insurance companies, etc.). This model of average wage earners generates a pervasive model of *wage-based* middle class society.
2. In Galbraith’s (1958) model of the *affluent society*, the standard of living increases over the life course leading to increasing levels of consumption as well as savings (in particular in home ownership). The wealth-to-income ratio is low and the median earnings are sufficient for enjoying comfort, which is a new feature compared to former societies, where wealth was the nodal resource. This model promotes equality through wage moderation at the top of the distribution (Fourastié 1979), in a context of sustained rapid economic growth where no social strata is deeply frustrated.
3. Welfare state development complements the protection provided by the permanent wage earner contract (lower volatility) extending thus *social citizenship*. Major social risks (widowhood, retirement, health, unemployment, old-age poverty etc.) are better covered by developed social insurance regimes (Ferragina and Seeleib-Kaiser 2011; Schröder 2019). In this model, *social protection* is a form of *depatrimonialisation*: wealth ownership is no more a condition of predictability.
4. Galbraith underlined the specific role of education, not only for obtaining selective skills that define the “new middle class” by comparison to the old one based on petty property, workshop, and boutique, but also for values and identity of middle-class parents who measure their own social success based on the educational performance of their children (i.e., entry ticket to upward social mobility). Thus, a middle-class society is characterized by a growing, publicly funded tertiary education sector (“educational boom”), able to offer younger generations greater human capital as the stepping-stone for *upward social mobility* and thus fostering the *belief in a meritocratic society*.
5. In the 1970s, values of socioeconomic progress and an optimistic vision of a never-ending search of personal and collective improvement in human development as well as economic, technological, and scientific progress characterized middle-class societies. In the American history of the middle class, the late 1960s were the climax of the *belief in progress* (“Man on the Moon”).
6. In the context of the post-war *Golden Age* (US/UK), *Miracolo economico* (Italy), *Rekordåren* (Sweden), *Wirtschaftswunder* (Germany), or *Trente glorieuses* (France) (Fourastié 1979), the middle class became an increasingly powerful political force. Traditional politics were based on the fight between the dominant bourgeois powers and the social critique of proletarian streams. Trade union forces were initially devoted to the defense of working class interests, not the median wage earner. In this political model, the middle class had in many countries a very limited political choice and often joined the bourgeoisie in right wing voting. Later, with its increasing size, the middle class gained *political centrality* in democratic elections (the so-called median voter). In the context of the post-1968 social movements, trade unions had been able to include large fractions of the public sector new middle class, in particular in Continental Europe. By the late 1980s, unions in the Scandinavian countries had widened their influence to include large segments of the growing middle strata (Marklund 1988).
7. Middle-class values in a middle-class society fit with the Aristotelian ideal of moderation, stability, and rationality. Due to progressive change in the context of post-materialist societies, the older political balance between proletariat and bourgeoisie gave way to the promotion of the middle class as a *centered moderated actor*, as prophesized by Simmel or Bernstein.

These seven parameters, typical of the *Golden Age* period of equalitarian expansion in Western countries, can generate a core of centripetal forces typical of middle-class societies. They are not only defined by a large proportion of middle-class members, but rely also on the consciousness of bourgeoisie and working class that their own social destination (or their children) is in the middle class. The centripetal forces are typical of the 1970s’ spirit where even non-middle-class actors, in the skilled working class and elsewhere, share some of the new middle-class interests (Kocka 1981).

## Diagnosis of middle-class societies: are there symptoms of a destabilizing social class?

The inversion of Galbraiths’ seven parameters is typical of centrifugal dynamics from the middle class. The destabilization of the former middle class trends gain in importance in periods of economic retrenchments and might produce a generation of young adults marked by a pessimistic *Zeitgeist* (spirit of the time, Mannheim 1928). The outline here is more programmatic than a definitive demonstration as that would require more systematic, comparative, long-range validation. The main claim here, however, is that elements of social destabilization that concerned the working class are climbing the socioeconomic ladder and reach at least the lower middle class. This section systematically re-assesses the seven arguments presented above to test the hypothesis of “a destabilizing middle class”: do we witness a general reversal of the seven trends in the post-*Golden Age* period and thus a decay of the middle class society?

1. Loss of stability in careers and fluctuations in the labor market generate wage uncertainty and thus difficulties to make plans for the middle class. A new massive precariat (Standing 2011) emerges in middle-class societies, particularly among younger generations (Mayer 2009). This status uncertainty includes new risks of over-indebtedness and vulnerability (Russell et al. 2012; Ahlquist and Ansell 2017). One of the strongest transformations of the middle class is its relation to security, in terms of lifelong control of adverse events. The security of a permanent job, or sufficient and scheduled working hours, is a central goal for the majority of the population, one which has been achieved by masses of wage earners in the 1960s, early on in their lifetimes. In the American case, increasing vulnerability of large segments of the lower middle class (Newman 1988; Newman and Chen 2007) become an obvious threat for the children of the Golden Age middle class. The consequences in terms of health, anticipated by Therborn (2013), have been extensively documented recently, where “deaths of despair” are the conclusion of increasing *collective insecurity* (Case and Deaton 2020).
2. The slowdown in economic growth negatively affects wage earners, even in “affluent societies.” The *income stagnation* is even clearer when we consider net wages, after payroll and income tax. Globalization (Milanovic 2016), market competition between continents, and automatization (Autor 2015) accelerate this trend. Moreover, we observe an increasing gap between economic growth (GDP per capita) and median net wages (after tax) which stagnates in many countries (Nolan et al. 2016). These trends differ by Welfare regime but accompanying a superficial upgrading of native men and women’s by occupational employment is a destabilization of their ability to rely on a lifetime of middle-class living standards (permitted by a capacity to avoid overindebtedness, buy property, have guaranteed hours, pensions, wages, permanent contracts) (Oesch 2015; Chauvel and Bar-Haim 2016).
3. A model of wage earner protections facing welfare state retrenchments, and the erosion of public insurance or its replacement by private insurances, wreaks havoc on household incomes—even in Nordic countries (Farrants and Bambra 2018). Targeted and means-tested welfare regimes progressively exclude the middle class from social protection: the poorer being protected and the richer able to afford their own needs on the market, the median being too rich to be protected and too poor to be dominant in market competition. As a consequence, savings, business resources, and capital gains make an increasing difference in individuals’ protection, where wealth accumulation, not social contributions to collective insurance systems, forms the major source of personal protection against risks. This means a large trend reversal after a complete twentieth century of Welfare State construction and decommodification described by Esping-Andersen (1990), in a new trend of *recommodification and return to market-based provision of “social protection”* (Schrecker and Bambra 2015) and thus *rewealthization*. This destabilization opens up new vulnerabilities over the entirety of the life course (Spini et al. 2017).
4. In several countries (e.g., Italy, Spain), even the highly educated face difficulties in entering the labor market, generating a *mismatch between education and socioeconomic positions,* also known as *overeducation* (e.g., *nimileuristas* in Spain). Beliefs in the intrinsic value of mass education erode and middle-class members become conscious of *risks of sudden social downward mobility* (Attewell and Newman 2010). This is neither specific to Southern Europe nor the lower middle class—in countries like Great Britain, a tertiary level degree holds no more protective power against episodes of being Not in employment, education or training (NEET) in young adulthood than finishing school does (Platt 2007; Holmes et al. 2019).

Characterized by 40 years of increased (bar cyclical) unemployment rates, France provides an interesting illustration of this process of decline in the predictability of wage earner status. On the one hand, we can claim education is more and more protective, relatively, against unemployment since the gap between the educated population and the less educated has grown over time. On the other hand, in this process of acceleration of inequalities, diplomas lose their absolute protective power. In this respect, education is becoming a more necessary and less sufficient resource (Bar-Haim et al. 2019). This contributes to the long-term development of uncertainty and malaise in the wage earner society, in particular in the young generation (Karonen and Niemelä 2019; Yeung and Yang 2020).

5. In Europe, declining trust in the European Union construction and, in America, the increasing difficulties in the promotion of interpersonal trust and civil society participation (Skocpol 2000; Putnam 2007) provide an impression of *declining belief in the future, progress, and science*. In Geiger’s model, economic degradation, downward mobility of circumstances, and the lack of a reliable and stable regulating frame generate fear, frustration, and social disorganization. A strong core of shared values and sense of solidarity can limit centrifugal trends but when they are absent, societies face the risks of anomie and social unrest, trends typical of the French 2018 “yellow vest” movement of frustrated downward mobile individuals of the lower middle class (Chauvel 2019). We will not elaborate on this in detail here but the Covid-19 events provided Western population with *new worries* such as the feeling that even Western science is no longer able to solve emergent issues.
6. Declining participation in the institutions of social democracy, in particular in trade unions, marks the *loss of political centrality* of the middle class (Chauvel and Schroeder 2017). This comes with a trend of elitization of politics and of politicians in a winner-take-all process of political inequalities (Hacker and Pierson 2010; Jensen and van Kersbergen 2017) excluding the poor and the middle classes as well.
7. Problems that were previously limited to socially excluded groups or the working class now spill over to the lower middle class. *Populist parties* progressively succeed in gaining votes in the middle class, for instance the Front National in France or the FPÖ in Austria (e.g., Pastor and Veronesi, 2018). Western countries including France, Italy, Hungary, and the Netherlands face disquieting drifts from their democratic ideals (a recent example would be the mainstreaming of far-right political messages by way of a steady incorporation of rightwing agendas into prominent parties in the Netherlands Witteveen 2017). In France, the “Yellow Vest Movement” exemplifies anomic trends and populistic temptations in the lower-middle-class fractions experiencing downward mobility (Chauvel 2019).

## Conclusions: lasting consequences

The trend of rewealthization in Western countries, shared with some Eastern-Asian countries, is particularly difficult in the West since it is not mitigated by the massive economic acceleration that in particular China has enjoyed in the last generation (Li 2013; Li 2014). This notion is important and needs to be understood (i) in its dimension of reconstitution of wealth as a potentially massively asymmetric resource between the haves and the have-nots, (ii) in its relations with the reconstitution of family dynasties of assets controllers, and (iii) due to the asymmetric political power relations benefiting those who possess wealth, there are new potentialities of privatization of public resources in the interests of the wealthiest classes.

The former dynamic of “dewealthization” that culminated in the decades after World War II was driven by declining housing costs, the reduction (or even the marginalization) of private wealth as a source of economic power, the correlative expansion of wage as resource, and the increasing role of the State in strategic economic sectors that were previously managed under a traditional mode of capitalist control. In France, important sectors, such as railways, strategic industries (mining, energy production, automotive industry, etc.), and even banks, were typically nationalized in a context where the owners (individuals and families) of these former private companies accepted to take over a prominent political influence and role in the current public affairs, in search for more citizenship honor and less economic affluence. In the French experience, the most visible transformation of the French central bank *Banque de France* that was the private property of old bourgeois and aristocratic families (“*Les deux cents familles*”, the two hundred families who owned the central bank), a system that collapsed in the reforms of 1936 and disappeared with its nationalization of 1945 in the public Central Bank of France. This aspect of private money gaining exorbitant recognition in the (im)balance of public power can evoke a modern counterpart of Max Weber’s concept of patrimonialism, when public institutions become hereditary family property.

The process of rewealthization is a backlash dating back to the 1990s (earlier or later depending on the country) when we observed the formation of a gap between pure wage earners, even with competitive credentials, diploma, and marketable skills, who are structurally unable to become homeowners, and wealth accumulators. This new structure of socioeconomic power reconfigures the middle classes: the new divide improves the relative position of seniors (juniors can *become* wealth accumulators, but have difficulties to *be* before age 50, in a demographic regime of high life expectancy) and especially reconstitutes the relative socioeconomic power of wealthy families over the others. Another aspect which we have not covered in this paper, but for which there are signs of an inverted order of older inequalities that could be morphing into new inequalities is a gender differentiation in WIR. In the welfare regime long touted for progressive gender egalitarian laws and labor market, Sweden, there are indications in top income dynamics that as increasing numbers of women now reach the top 1% of earners on the back of their labor incomes, they have dropped from 18 to 17% of the top group were wealth (capital) taken as their only resource. The role of wealth (capital, and realized gains) has switched from being more important for women than for men in the 1970s, to being the most important resource, and source of growth, for men not women by 2017 (Boschini et al. 2017). Many pieces of the wealth puzzle remain hidden, which future research might address by means of simulations based on known distributions and measured trends, to better quantify the expected divide within the middle class between those who own and those who do not.

The present diagnosis rests upon the systematization of observations in Western countries in the last three decades before the Covid-19 PANDEMIE, CONFIRMING THE MIDDLE CLASS CRISIS. In this period, China escalated the ladder to the opposite direction with the expansion of a stronger middle class (Li 2014), even if trends of rewealthization are debated (Xie and Jin 2015; Piketty et al. 2019; Li and Fan 2020). The reactions to the Covid-19 outbreak underlined once again the frailty of Western societies in terms of public health and problems of social consensus and resilience. This frailty encompasses responses to social challenges expressed by two scenarios of inequality. One scenario is of a recovery in the context of a more balanced growth based on policies of inequality reduction (Atkinson 2016). The opposite one is of an acceleration of previous Western social challenges: recession on several indicators of human development, and a radical divide between the haves and the have-nots to reconstitute the extreme inequality structures of the nineteenth century (Chauvel 2019; Case and Deaton 2020).

Even if European societies continue to define themselves as olive-shaped, debates emerge there on the accurate description of the new structure, which might be more rigid than the “圭(gui)-shaped” one (Liu 2020): for the Western middle-class, long-term economic slowdown reduces opportunities of structural social mobility. Abundance at the pinnacle of Western society could stand for scarcity for the rest, with stagnation and frustrations contributing to a potentially unstable equilibrium. This trend brings us far from a harmonious society of modest wealth.

## Supplementary Information

**Additional file 1:** Stata do-file to generate WIR and TWIR figures.

## Data Availability

Data are public; codes are available upon request to authors.

## Abbreviations

TWIR: Top wealth-to-average income ratio
WIR: Wealth-to-income ratio
WID: World Inequality Database
NEET: Not in employment, education or training
